# Muscle biopsies in children—an evaluation of histopathology and clinical value during a 5-year period

**DOI:** 10.1080/03009730802604949

**Published:** 2009-02-04

**Authors:** Marius Kurås Skram, Sasha Gulati, Erik Larsson, Sigurd Lindal, Sverre Helge Torp

**Affiliations:** ^1^Department of Laboratory Medicine, Children's and Women's Health, Faculty of Medicine, Norwegian University of Science and Technology (NTNU), Trondheim University HospitalTrondheimNorway; ^2^Department of Neurosurgery, St. Olavs HospitalTrondheimNorway; ^3^Department of Pathology, University HospitalTromsøNorway; ^4^Department of Pathology and Medical Genetics, St. Olavs HospitalTrondheimNorway

**Keywords:** Biopsy, diagnosis, dystrophy, electron microscopy, immunohistochemistry, myopathy, neuromuscular, paediatrics, pathology, skeletal muscle

## Abstract

Muscle biopsy is an important diagnostic tool in the investigation of children with neuromuscular disorders. This report presents the experience with paediatric muscle biopsies during a 5-year period at a routine pathology laboratory. A total number of 58 cases were included, and indications, microscopical findings, and final histopathological diagnoses were recorded. A total of 21 biopsies were from females (36%) and 37 biopsies from males (64%); 53% of the cases were from children under 2 years of age. Major pathological findings were found in 30% comprising muscular dystrophy, neurogenic atrophy, and congenital and metabolic disorders, even in cases with vague clinical manifestations. These findings confirm the high diagnostic yield of muscle biopsies, especially as new techniques have been introduced such as immunohistochemistry. Muscle pathology is difficult and emphasizes the importance of this service being undertaken by specialized laboratories with an experienced staff. Microscopical examination of muscle biopsies should be based on adequate clinical information, demonstrating the necessity of close contact between pathologists and referring physicians.

## Introduction

Investigation of a child with suspected neuromuscular disorder is challenging, and muscle biopsy represents an important diagnostic tool. New techniques, such as immunohistochemistry, have improved the diagnostic value of this procedure (1–4).

Obtaining a muscle biopsy, specimen handling, and microscopic examination are time-consuming and resource-demanding (5). Evaluation of this procedure with special focus on resource management and quality assurance is therefore of great value. Furthermore, the clinicians may have a wish for an insight into this procedure.

The aim of this study was therefore to review and evaluate paediatric muscle biopsies during a 5-year period with focus on indications, histopathology, analyses, diagnoses, and utility value.

## Materials and methods

All muscle biopsies in Mid-Norway are examined at the Department of Pathology and Medical Genetics, St. Olavs Hospital, Trondheim University Hospital, Trondheim, Norway. The hospital serves three counties with a total population of about 650,000 people, of whom approximately one-fourth is under the age of 18. Muscle biopsies are performed both at district general hospitals and at the university hospital. Needle biopsy using local anaesthesia is the preferred technique; however, sometimes an open muscle biopsy is carried out under local or general anaesthesia. Outside the university hospital the specimen is kept cool on ice, transported to the pathology department the same day, either by train or plane. If the muscle biopsy is performed at the university hospital, it is sent to the laboratory immediately after removal, kept dry in a test tube.

The computer-based patient data files at the laboratory were searched for muscle biopsies during the time period 1998–2002. A total number of 281 biopsies were found, of which 58 cases (21%) were from patients younger than 19 years of age. These muscle biopsy reports were reviewed, and the following data were recorded: age, gender, indication for biopsy, results of microscopical findings, histochemistry, enzyme histochemistry, immunohistochemistry, electron microscopy, and histopathological diagnosis.

The muscle biopsy is prepared for light and electron microscopy. Both paraffin and frozen sections are cut routinely; the latter are especially suitable for enzyme histochemistry and immunohistochemistry. General histochemistry comprises haematoxylin/eosin, Gomory trichrome (detection of ragged-red fibres and nemaline rods), oil red (detection of lipids), and periodic acid-Schiff (PAS) (detection of glycogen). Enzyme histochemistry demonstrates endogenous enzymatic activities in muscle fibres and is used to analyse different fibre types, detection of subcellular organelles, structural abnormalities, and specific enzymatic defects. Immunohistochemistry is used for identification of proteins and cellular elements, for instance to characterize cells in inflammatory myopathies (B cells, T cells, macrophages) and to determine fibre types, dystrophin, and dystrophin-associated proteins. Investigation of muscular dystrophy sometimes requires Western blotting. Electron microscopy is frequently performed in paediatric muscle biopsies, especially when metabolic, mitochondrial, and congenital disorders are suspected, including nemaline myopathy, myotubular myopathy, and central core disease. In difficult cases biopsies are sent to the National Competence Centre for Muscle Disease, University Hospital, Tromsø, Norway, for consultation.

## Results

In this series of 58 paediatric muscle biopsies no major artefacts were observed after dispatching or processing that might have prevented an adequate microscopical evaluation.

Our pathology department receives annually about 75 muscle biopsies, of which paediatric ones comprise 10–15 specimens. The female:male ratio was 0.6. More than half of the patients (53%) were below 2 years of age, 17 patients (30%) between 3 and 9 years, and 10 patients (17%) in the age-group 10–18 years.

Indications for obtaining muscle biopsies were recorded according to a modification of Heffner and Schochet (6). Corresponding histopathological diagnoses were listed as well. Out of 58 cases, 22 (38%) underwent a muscle biopsy intervention due to various muscle symptoms (pain, cramps, stiffness, and paraesthesia), and 16 of them (73%) revealed normal skeletal muscle or unspecific changes. The remaining 6 cases revealed specific entities including neurogenic atrophy (2 cases), muscular dystrophy (not other specified) (2 cases), mitochondrial myopathy (1 case), and congenital myopathy (central core disease) (1 case). Weakness or hypotonia were indications in 11 out of 58 cases (19%), of which 8 cases demonstrated no pathology or unspecific changes. In the remaining three cases congenital disorders were detected (one congenital myopathy and two cases with nemaline myopathy). Metabolic disease was suspected in 10 cases (10/58, 17%); all showed normal skeletal muscle or unspecific changes. Questions of muscular dystrophy were raised in 9 cases (9/58, 16%), and dystrophic changes were seen microscopically in 4 cases (Duchenne muscular dystrophy (3 cases) or Becker muscular dystrophy (1 case)), mitochondrial myopathy (1 case), and neurogenic atrophy (1 case). Three cases with elevated serum creatine kinase values displayed no histopathological changes. In two cases with suspected inflammatory myopathy, one disclosed dermatomyositis and one connective tissue disease.

Normal skeletal muscle was found in about half of the cases (28/58, 48%). Unspecific or chronic myopathic changes, defined by Cumming et al. (7) as atrophy, hypertrophy, internal nuclei, necrosis, regeneration, fibrosis, and splitting, were encountered in 13 cases (22%). Four out of six cases with dystrophic changes were found to represent Duchenne or Becker type due to lack of or reduced immunostaining for dystrophin; it was not possible to classify the two remaining cases. Neurogenic atrophy was seen in three cases, one was Werdnig-Hoffmann disease and one with neuroaxonal dystrophy. There were two cases with myositis consistent with dermatomyositis and one case with muscular involvement as a facet of connective tissue disorder. Ragged-red fibres were found in two cases consistent with mitochondrial myopathy. Four cases turned out to be congenital muscular disorders: nemaline myopathies (2 cases), central core disease (1 case), and one could not be classified. Table I shows cases with major pathological findings.

**Table I.** Survey of cases with major pathological findings and diagnoses.^a^

Age (years)	Sex	Indication for muscle biopsy	Major pathological findings	Supplementary analyses	Final diagnosis^a^
0	M	Hypotonia	Chronic myopathic changes	Glycogen deposits (EM)	Congenital myopathy possible
0	M	Muscle pain, cramps, stiffness, etc.	Neurogenic atrophy		Spinal muscle atrophy consistent with Werdnig-Hoffmann disease
0	F	Hypotonia	Nemaline bodies	Nemaline rods (EM)	Nemaline myopathy
1	M	Muscle pain, cramps, stiffness, etc.	Dystrophic changes and cores	Myofilament pathology (EM)	Central core disease
2	M	Muscle pain, cramps, stiffness, etc.	Neurogenic atrophy		Neurogenic atrophy (neuroaxonal dystrophy)
2	F	Hypotonia	Nemaline bodies	Nemaline rods (EM)	Nemaline myopathy
2	F	Dermatomyositis?	Chronic inflammation and atrophy	B and T lymphocytes (IHC)	Inflammatory myopathy consistent with dermatomyositis
2	M	Muscular dystrophy?	Dystrophic changes	Lack of dystrophin (IHC)	Duchenne muscular dystrophy
3	M	Muscular dystrophy?	Neurogenic atrophy	Dystrophin-associated proteins present (IHC)	Neurogenic atrophy
3	M	Muscle pain, cramps, stiffness, etc.	Dystrophic changes	Dystrophin-associated proteins present (IHC)	Slowly progressive primary myopathy consistent with muscular dystrophy
4	M	Muscular dystrophy?	Dystrophic changes	Lack of dystrophin (IHC)	Duchenne muscular dystrophy
5	M	Muscular dystrophy?	Dystrophic changes	Lack of dystrophin (IHC)	Duchenne muscular dystrophy
5	M	Muscle pain, cramps, stiffness, etc.	Dystrophic changes	Dystrophin-associated proteins present (IHC)	Slowly progressive primary myopathy consistent with muscular dystrophy
6	M	Muscular dystrophy?	Ragged-red fibres	Abnormal mitochondria and deposits of lipid and glycogen (EM)	Metabolic myopathy, mitochondrial myopathy possible
9	F	Muscle pain, cramps, stiffness, etc.	Ragged-red fibres	Abnormal mitochondria and deposits of glycogen (EM)	Metabolic myopathy, mitochondrial myopathy possible
10	M	Muscular dystrophy?	Dystrophic changes	Partial lack of dystrophin (IHC, WB)	Becker muscular dystrophy
16	F	Inflammatory muscle disease?	Necrosis		Necrotizing myopathy, probably in association with connective tissue disease

^a^Classification modified after (8). EM = electron microscopy; IHC = immunohistochemistry; WB = Western blotting.

**Figure 1.**
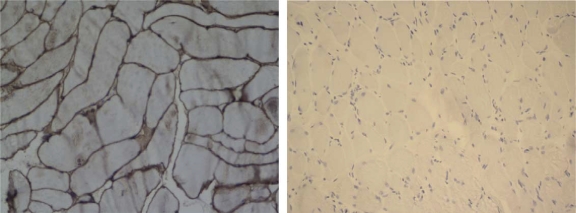
Muscle biopsy specimen with immunostaining for dystrophin: normal expression (left) and absence of expression in a case of Duchenne muscular dystrophy (right).

**Figure 2.**
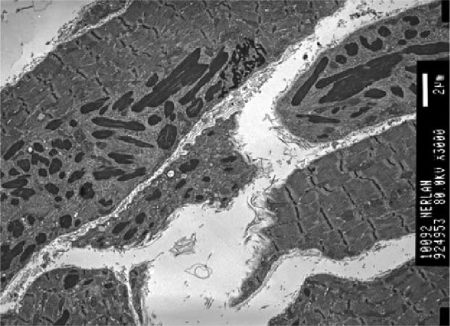
Electron micrograph of nemaline rods.

Auxiliary techniques such as immunohistochemistry were frequently requested (74%) in order to achieve a more distinct diagnosis, as illustrated by lack of dystrophin expression (Figure 1). Electron microscopy was performed in 24 out of 58 biopsies (41%) and demonstrated important ultrastructural pathology in 8 cases showing abnormal mitochondria, deposits, or nemaline rods (Figure 2). In 23 cases (40%) the centre of competence was consulted, for the most when muscular dystrophy was suspected. In such cases extended immunohistochemical analyses were performed with a battery of antibodies against dystrophin-associated proteins, often supplemented with protein blotting. Sometimes genetic analyses were recommended, especially in patients under investigation for mitochondrial disorders and muscular dystrophies.

## Discussion

This report deals with the experience with paediatric muscle biopsies in the daily routine at a general pathology department during a 5-year period.

All muscle biopsies taken in Mid-Norway are analysed at the Department of Pathology and Medical Genetics, St. Olavs Hospital, Trondheim, Norway, and close contact has evolved between pathologist and referring physicians providing relevant clinical information for optimal diagnostic histopathology. Thus, one consultant pathologist is dedicated to this service since muscle pathology has become highly specialized. In fact, collaboration with centre of expertise is requisite, and several biopsies are sent to The National Competence Centre for Muscle Disease at the University Hospital in Tromsø, Norway, for consultation. This laboratory is equipped with experienced scientific and medical staff that provides more sophisticated and extensive services and investigations, including advanced immunohistochemistry with a large panel of antibodies, as well as Western blotting and molecular genetic analyses.

Histopathological changes in muscular disorders are often unspecific, and clinical information is the prerequisite of making good diagnoses. In each case, however, the pathologist should try to identify a diagnostic category, such as normal, unspecified, dystrophic, inflammatory, etc., since the biopsy report serves as therapeutic guideline for the clinician; even exclusion of a diagnosis is important (5).

Indication for performing muscle biopsy is in general wide, and in the present study the most common cause was various muscular symptoms and signs, including pain, cramps, stiffness, paraesthesia, weakness, and hypotonia (57%). The high number of cases under investigation for metabolic (17%) and hereditary disorders (including muscular dystrophy) (17%) was not unexpected. Among the first group normal muscle tissue or chronic myopathic changes were found, whereas in the latter specific pathology was recorded in 6 cases (Duchenne muscular dystrophy (3 cases), Becker muscular dystrophy (1 case), neurogenic atrophy (1 case), mitochondrial myopathy (1 case)). Thus, the clinical yield of muscular biopsy is fruitful as this procedure may unveil specific disorders despite sparse clinical manifestations.

Since several biopsies (41/58, 71%) displayed normal skeletal muscle tissue or unspecific changes, one may ask whether the indications are too liberal. Performing a muscle biopsy is painful, and full anaesthesia may be necessary. Accordingly, the physician should consider the indications for muscle biopsy carefully. On the other hand, investigation of children with neuromuscular disease is challenging, and this study has clearly confirmed the clinical value of a muscle biopsy, defending a liberal attitude to this procedure. Needle biopsy is nowadays the preferred method as it is less time-consuming, more cost-effective, and encumbered with fewer complications than an open biopsy. Disadvantages are small and often traumatized tissue samples, although we did not experience troublesome artefacts in this series of muscle biopsies.

Supplementary analyses, such as immunohistochemistry, histochemistry, and electron microscopy, have resulted in identification of new pathological structural changes (1–4,9), clearly illustrated by ultrastructural findings in cases of congenital and metabolic myopathies, and immunostaining in cases of muscular dystrophies and inflammatory myopathies. In this manner the utility value of a muscle biopsy has been greatly improved, providing more accurate and specific diagnoses. Nevertheless, advances in molecular genetic analyses have led to identification of diverse gene defects. For this reason, in patients with a family history or with a phenotype suggesting a well characterized hereditary disorder, available genetic tests on a blood sample or of any other tissue should be considered (4,5).

In conclusion, muscle biopsy is invaluable in the investigation of children with neuromuscular disorders, especially by implementing auxiliary techniques such as immunohistochemistry and electron microscopy. Histopathological examination is, however, time-consuming and requires experienced personnel, so co-operation with a skills centre is necessary. Further, close liaison between pathologist and clinicians is essential, and histopathological findings should only be interpreted in the light of clinical manifestations and laboratory findings. Finally, a study like this represents a useful evaluation of this medical procedure and is important in the quality assurance routine of the pathology department.
